# Radiotherapy De-Escalation in Younger Patients with Breast Cancer: Are We There Yet?

**DOI:** 10.3390/cancers18040639

**Published:** 2026-02-16

**Authors:** Ioannis Georgakopoulos, Georgios Nikiforos Ntoumas, Pantelis Skarlos, Alexia Sidiropoulou, Georgia Lymperopoulou, Ioanna Kollarou, Konstantina Perdikari, Flora Zagouri, Maria Tolia

**Affiliations:** 1 Radiation Oncology Unit, 1st Department of Radiology, Medical School, Aretaieion Hospital, National and Kapodistrian University of Athens, 11528 Athens, Greece; geontoumas@yahoo.com (G.N.N.); alesidiro1@gmail.com (A.S.); glymper@med.uoa.gr (G.L.); 2 Radiation Oncology Department, General University Hospital of Larissa, 41334 Larissa, Greece; pskarlos@uth.gr; 3 Medical Oncology Unit, Medical School, Aretaieion Hospital, National and Kapodistrian University of Athens, 11528 Athens, Greece; joanna.collaros@gmail.com (I.K.); konna_perd@hotmail.com (K.P.); fzagouri@med.uoa.gr (F.Z.); 4 Radiation Oncology Department, University General Hospital of Heraklion, 71500 Heraklion, Greece; mariatolia@uoc.gr

**Keywords:** breast cancer, young patients, radiotherapy, de-escalation

## Abstract

**Background/Objectives**: Radiotherapy de-escalation is an established strategy in the management of early breast cancer, supported by randomized evidence predominantly derived from older patient populations. Younger women remain underrepresented in de-escalation trials, despite exhibiting less favorable clinicopathological characteristics associated with increased locoregional recurrence and inferior survival. The objective of this systematic review is to assess the available evidence regarding the safety and implementation of radiotherapy de-escalation strategies in younger patients with early breast cancer. **Methods**: A literature search following the PRISMA 2020 guidelines was performed to identify studies evaluating radiotherapy de-escalation strategies in younger breast cancer patients. Ongoing and recently completed trials were identified through ClinicalTrials.gov. Epidemiological data, randomized trials, and current clinical guidelines were reviewed. **Results**: Younger age at diagnosis is consistently associated with more aggressive tumor biology, higher rates of nodal involvement, unfavorable molecular subtypes, and worse survival outcomes. Among de-escalation approaches, moderate hypofractionation (15–16 fractions) is supported by randomized evidence and contemporary guidelines and can be applied irrespective of age. In contrast, evidence supporting ultra-hypofractionation, partial breast irradiation, and omission of radiotherapy in younger patients remains less robust, as these strategies have largely been evaluated in older or postmenopausal populations. **Conclusions**: Radiotherapy de-escalation in younger patients with breast cancer should be approached with caution. While moderate hypofractionation appears safe regardless of age, more aggressive de-escalation strategies lack adequate evidence in women under 50 years, particularly those under 40. Further prospective studies with sufficient representation of younger patients are required to clarify the role of radiotherapy de-escalation in this population.

## Simple Summary

Radiotherapy de-escalation is an important approach in the management of early breast cancer, with the goal of limiting treatment burden, without compromising oncologic outcomes. However, implementation of such strategies in younger patients remains precarious, not only due to their underrepresentation in relative studies but also due to the more aggressive tumor biology, higher recurrence risk and worse survival observed in younger individuals. This review summarizes existing data on radiotherapy de-escalation strategies specifically in younger women with early breast cancer. The evidence supports that moderate radiotherapy hypo-fractionation can be safely applicated irrespective of age, as opposed to ultra-hypofractionation, partial breast irradiation and omission of radiotherapy, for which data remain uncertain. Future trials with adequate representation of younger patients are required to clarify the safety and applicability of de-escalation approaches to this particular population.

## 1. Introduction

The contemporary trend in adjuvant breast radiotherapy is treatment de-escalation [1]. This may include shortening the overall treatment duration through hypofractionation, modifying treatment fields through partial breast irradiation or selective irradiation of regional lymph nodes, or, finally, omitting radiotherapy altogether in selected low-risk patients [2].

Although evidence exists for older age groups and some of these data are sufficiently mature to have been incorporated into clinical guidelines, due to the underrepresentation of younger patients in relevant randomized trials, we are not yet in a position to state with confidence that all de-escalation and hypofractionation protocols can be safely applied in younger women [3].

The aim of this article is to review the available literature in order to assess whether radiotherapy de-escalation can be implemented in younger patients diagnosed with breast cancer or whether additional evidence from ongoing and future studies is still required.

## 2. Materials and Methods

A comprehensive search of the published literature was conducted using the PubMed, Embase and Cochrane library databases to identify studies regarding the use of de-escalation treatment strategies in younger patients with breast cancer. We employed relevant terms, including “breast cancer”, “younger patients”, “radiotherapy”, “de-escalation” and implemented Boolean operators to create the following core search string: (“breast cancer” OR “breast carcinoma”) AND (“radiotherapy” OR “radiation therapy”) AND (fraction* OR hypofraction* OR “whole breast irradiation” OR “partial breast irradiation” OR “breast irradiation”). Only articles published in English were included, and no restrictions regarding study design or country of origin were applied. The final search was conducted in December 2025. Because many pivotal breast radiotherapy trials do not explicitly reference age or younger patients in titles or abstracts, age-related terms were not applied as mandatory database filters. Instead, age eligibility criteria and age-specific representation were assessed during the screening and full-text review phases. To ensure further inclusion of trials focusing on de-escalation and omission of radiotherapy, a supplementary search was performed with the following string: (“breast cancer”) AND (“radiotherapy”) AND (“ultra-hypofractionation” OR “partial breast irradiation” OR “radiotherapy omission” OR “de-escalation”). We also searched the ClinicalTrials.gov database to identify ongoing relevant studies. Trials were included if they explicitly enrolled patients below 50 years of age or reported age-stratified eligibility criteria, investigated partial-breast irradiation, hypofractionation, or omission of radiotherapy, and were actively recruiting or had not yet reported primary results. For each trial, we extracted the following data: trial identifier (NCT number), enrollment target, age eligibility cutoffs, primary endpoint(s), and estimated primary completion date. This approach enables a transparent assessment of evidence gaps and the anticipated timing of results relevant to younger cohorts.

**Figure 1:**
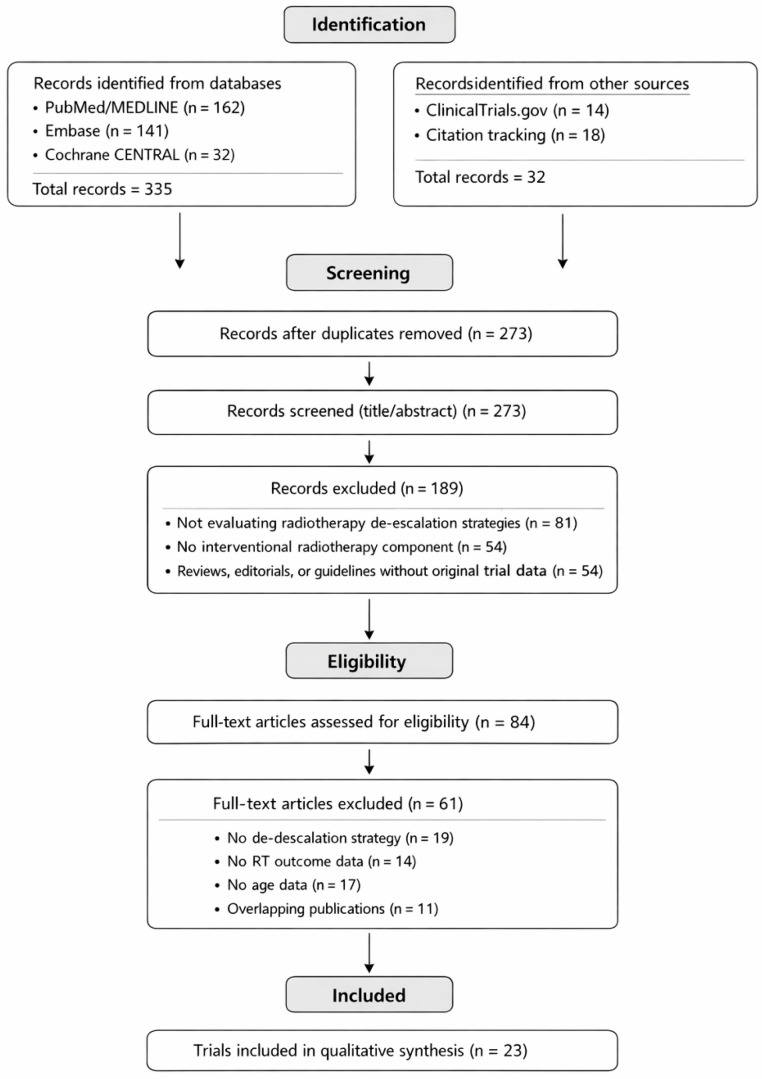
PRISMA 2020 flow diagram of study identification and selection.

The PRISMA 2020 flow diagram is illustrated in Figure 1. The PRISMA 2020 Checklist is list in Supplementary Materials.

For randomized controlled trials evaluating radiotherapy de-escalation strategies, risk of bias was assessed qualitatively using domains consistent with the Cochrane Risk of Bias 2 (RoB 2) framework, including randomization process, deviations from intended interventions, missing outcome data, outcome measurement, and selective reporting.

The overall certainty of evidence for each de-escalation strategy was summarized using GRADE-style terminology (high, moderate, low, or very low), based on study design, consistency of results, directness of evidence, and applicability to younger patients. Particular emphasis was placed on age-specific representation, selection criteria, and potential confounding from systemic therapy.

This review was prospectively registered in the Research Registry (reviewregistry2073). The protocol prespecified the objectives, inclusion/exclusion criteria, data extraction items, and planned analyses. The review was conducted in accordance with the PRISMA 2020 statement, including structured literature searches, independent screening and extraction by two reviewers, and transparent reporting of results and risk-of-bias assessments.

## 3. Results

### 3.1. Epidemiological Data

According to epidemiological data from the USA, breast cancer is the most commonly diagnosed malignancy and the second leading cause of cancer-related mortality in the Western world, after lung cancer. Its incidence increases with age, as more than 72% of cases occur after the age of 55. However, a substantial proportion of approximately 28%, nearly one in three cases, occurs in women younger than 54 years. Specifically, the incidence is 18% among women aged 45–54 and 8.5% among women younger than 45 [4].

### 3.2. Distinct Disease Characteristics in Younger Patients

A particularly important and widely accepted observation, supported by robust evidence, is that breast cancer diagnosed at a younger age is associated with more aggressive clinical and pathological features compared with disease in older patients. In a study by Zabicki et al., patients younger than 40 years, compared with those aged 50–60, were diagnosed with larger primary tumors and had a higher likelihood of nodal involvement [5].

In terms of tumor biology, younger age has also been associated with a more aggressive phenotype. In a 2022 study analyzing data from SEER database including approximately 270,000 patients, diagnosis before the age of 40 was associated with a higher probability of more advanced stage disease (21.4% vs. 13.7% in patients aged 40–60), poor differentiation (54.9% vs. 34.8%), estrogen receptor negativity (71.3% vs. 18.7%), and triple-negative breast cancer (19.5% vs. 11.9%). In the same study, disease-specific survival was also analyzed, showing that the aggressive phenotype of younger patients had a clear negative impact on survival, which was significantly worse for patients younger than 40 compared with all age groups older than 40 [6].

In a recent study by Oshi et al. two cohorts from the METABRIC (Molecular Taxonomy of Breast Cancer International Consortium) database and a Scandinavian cohort were analyzed, for a total of 3734 patients. The ER-positive/HER2-negative subtype was evaluated in relation to patient age. Pathological lymph node positivity and grade 3 disease were more frequent among adolescents and young adults younger than 40 years (all *p* < 0.001). Young age was also associated with worse disease-specific and overall survival compared with the perimenopausal group, and with significantly higher rates of BRCA gene mutations than in other age groups (all *p* < 0.05 in both cohorts) [7].

### 3.3. Age-Specific Issues in Younger Patients

A key issue is the distinct psychological burden experienced by this patient population, as diagnosis at a younger age is associated with an increased risk of psychological distress, largely related to sexual dysfunction, concerns regarding menopausal status, and fertility issues. In a review study by Howard-Anderson et al., which included 28 articles (including five randomized trials), breast cancer in younger women was clearly associated with a substantial negative impact on quality of life, with symptoms of depression and anxiety, concomitant weight gain, and reduced daily activity [8].

### 3.4. Radiotherapy De-Escalation in Younger Patients

#### 3.4.1. Hypofractionation

Large, randomized trials have historically defined the optimal radiotherapy regimen for patients with an indication for adjuvant radiotherapy after mastectomy or breast-conserving surgery. Trials such as MILAN, NSABP B-06, and EORTC 10801 established conventional schedules of 25–28 fractions, delivered at 1.8–2 Gy per fraction, to total doses of 50–50.4 Gy. In the setting of breast-conserving surgery, a boost to the tumor bed typically followed, commonly to a total dose of approximately 60 Gy [9,10,11].

Since the early 2010s, well-designed randomized trials have compared “classic” conventional schedules with hypofractionated regimens, consisting of fewer fractions—typically 15 or 16 instead of 25—with higher daily doses (approximately 2.66 Gy vs. 2 Gy). Across these trials, the two regimens were shown to be therapeutically equivalent, and in some studies the hypofractionated approach demonstrated a more favorable toxicity profile. Specifically, no difference was observed in local control or survival, while trials that included acute and late toxicity endpoints—such as the MD Anderson study and the Canadian trial —showed superiority of hypofractionation over conventional fractionation in terms of toxicity [12,13,14,15,16,17].

Representation of younger patients in these trials was limited; however, younger women were clearly included in the randomization process. Approximately 25% of patients across these studies were younger than 50 years, and although age younger than 40 was not an exclusion criterion, the average proportion of patients younger than 40 was approximately 7%, representing a small subgroup with limited statistical power (Table 1). Despite the limited inclusion of young patients, age-specific subgroup analyses were reported. In the Canadian trial, with 10-year follow-up, the treatment effect on ipsilateral local recurrence with hypofractionation was similar irrespective of age, with reported HRs of 1.02 (CI 0.62–1.70) and 0.77 (CI 0.35–1.70) for patients ≥50 and <50 years of age [17]. Similarly, in the START trials’ pooled analysis, there were no differences in locoregional relapse and moderate or marked physician-assessed normal tissue effects across all age subgroups. Specifically, reported HRs were 0.79 (CI 0.47–1.34), 0.88 (CI 0.60–1.28), 1.03 (CI 0.74–1.44) and 1.11 (CI 0.75–1.63) for locoregional relapse and 0.85 (CI 0.56–1.28), 1.09 (CI 0.86–1.37), 0.78 (CI 0.68–0.91) and 0.80 (CI 0.69–0.92) for normal tissue changes, regarding patients <40, 40–49, 50–59 and ≥60 years of age, respectively [14].

#### 3.4.2. Ultra-Hypofractionation Treatment Protocols

More recent data, including randomized trials, have also evaluated ultra-hypofractionated regimens, using even higher daily radiation doses and completing treatment in only five fractions. The best-known trial is the FAST trial from the United Kingdom, which compared the conventional 25-fraction schedule with a five-fraction schedule delivered once weekly, at 5.7 or 6 Gy per fraction, to total doses of 28.5 or 30 Gy, respectively. The primary endpoint was cosmetic outcome at 2 and 5 years, with secondary endpoints including telangiectasia, breast shrinkage, edema, and local control. This trial demonstrated no meaningful differences between conventional and ultra-hypofractionated treatment, particularly for the 28.5 Gy arm. However, it included patients with favorable, low-risk breast cancer who were older than 50 years; therefore, conclusions cannot be drawn for younger age groups [18].

In the same year, the FAST-Forward trial, also from the United Kingdom, was published. This three-arm randomized trial compared a 15-fraction hypofractionated schedule with two five-fraction regimens delivered over five consecutive days (5.4 Gy or 5.2 Gy per fraction). A total of 4096 patients with low-risk breast cancer after breast-conserving surgery (pT1–3 pN0–1) were enrolled, with local control as the primary endpoint. Importantly, younger patients were eligible, from 18 years of age. The study showed non-inferiority, particularly for the 26 Gy regimen (5.2 Gy per fraction) compared with 40 Gy in 15 fractions, with a reported HR for ipsilateral breast tumour relapse of 0.67 (CI 0.38- 1.16) for 26 Gy in 5 fractions vs. 40 Gy in 15 fractions. Nonetheless, representation of younger patients was limited, with only 2% younger than 40 years and 13.8% aged 40–49, rendering subgroup analyses non-reliable. Therefore, robust conclusions for younger age groups cannot be drawn [19]. While extrapolation from START trials to ultra-hypofractionation should be made cautiously, the absence of age-related heterogeneity in moderate hypofractionation trials provides indirect reassurance that younger age alone does not appear to modify fractionation sensitivity [19].

#### 3.4.3. Hypofractionation in the Setting of Regional Nodal Irradiation

Despite the adoption of hypofractionated schedules in breast radiotherapy—especially after breast-conserving surgery—randomized evidence supporting shorter schedules when regional nodal irradiation is required had been limited. In 2024, the HYPOG-01 trial addressed this question by evaluating a hypofractionated regimen in patients requiring radiotherapy to regional lymph nodes after breast-conserving surgery or mastectomy. Endpoints included lymphedema, overall survival, progression-free survival, disease-specific survival, and shoulder mobility.

The trial clearly demonstrated non-inferiority of the three-week regimen, and investigators concluded that hypofractionation should now be considered the standard of care even when regional nodal irradiation is indicated. Importantly, younger patients were included, supporting the safe application of this approach irrespective of age [20,21].

#### 3.4.4. Hypofractionation in Younger Patients: Overall Summary

In summary, with the exception of ultra-hypofractionation delivered in five consecutive daily fractions, hypofractionated regimens have a clear indication regardless of age, a statement reflected in contemporary clinical guidelines. ESMO guidelines state that moderate hypofractionation (15–16 fractions) should now be routinely applied, while ASTRO guidelines explicitly support hypofractionation irrespective of age, reserving conventional fractionation (25–28 fractions) only for selected situations [22,23] (Table 2).

#### 3.4.5. De-Escalation of Radiation Fields

##### Partial Breast Irradiation

Studies from large surgical series have confirmed that in early-stage disease, the vast majority of recurrences occur in close proximity to the tumor bed. For example, Veronesi et al. reported that, in general, local recurrences were near the surgical bed, with fewer than 20% occurring elsewhere in the breast [24]. This supported the concept of partial breast irradiation (PBI) using external beam radiotherapy, where the irradiated volume focuses on the tumor bed—typically delineated by surgical clips—with an additional margin (commonly 2 cm circumferentially).

Early trials demonstrated mixed results, and a Cochrane meta-analysis reported unacceptably high local recurrence rates [25]. More recently, with optimal patient selection and inclusion of patients with very early breast cancer, evidence has emerged supporting a clear role for PBI. The Florence trial demonstrated non-inferiority of PBI, with identical local recurrence rates and a clear benefit in acute and late toxicity by avoiding irradiation of the whole breast. However, the minimum age for inclusion in this trial was 50 years, and therefore conclusions cannot be extended to younger patients [26]. Similarly positive results with comparable rates of local control and very low adverse effects but with age restrictions (>50 years), were reported in other trials such as IMPORT LOW [27].

Consequently, there are no robust data supporting the use of PBI in very young patients. The GEC-ESTRO trial, which evaluated PBI using multicatheter interstitial brachytherapy, included patients aged ≥40 years (15.4% of the cohort), but firm conclusions for younger ages remain limited [28]. This is reflected in clinical guidelines: ASTRO recommends PBI primarily for patients aged ≥50 years, while the American Brachytherapy Society suggests an age threshold of ≥45 years [29,30].

##### Selective Radiotherapy After Mastectomy

Recently, the SUPREMO trial reported its 10-year outcomes. This trial randomized intermediate-risk patients after mastectomy to receive chest wall irradiation or no radiotherapy. Eligible patients included those with pT1–2N1, pT3N0, or pT2N0 with grade 3 disease and/or lymphovascular space invasion (LVSI) positivity, who underwent mastectomy with nodal staging; in the presence of 1–3 positive lymph nodes, axillary clearance was required, with at least eight nodes retrieved.

The trial showed that omission of chest wall irradiation did not negatively affect endpoints, including no statistically significant differences in overall survival, local control, or metastasis-free survival. Investigators concluded that chest wall radiotherapy could be omitted in selected intermediate-risk patients, even with positive lymph nodes. The trial did not include an age criterion and enrolled younger patients; specifically, 15.6% of patients were younger than 45 in both arms.

However, the study has some limitations as only 12% of patients had three positive lymph nodes, only 0.4% had pT3 disease, and the cohort was heterogeneous regarding systemic therapy (including both neoadjuvant and adjuvant treatment) [31]. Therefore, based on current guidelines, such as the recent ASCO/ASTRO/SSO consensus, patients with node-positive disease generally have a clear indication for adjuvant radiotherapy [32].

##### Selective Irradiation After Neoadjuvant Systemic Therapy and Complete Nodal Response

Another important recent study is NSABP B-51, a phase III trial evaluating the benefit of adjuvant regional nodal irradiation in patients with early breast cancer who convert from node-positive to axillary node-negative status after neoadjuvant chemotherapy. The study demonstrated that in patients who achieve complete pathological nodal response, regional nodal irradiation may be omitted in selected patients with favorable disease characteristics.

This conclusion can reasonably be extended to younger patients, as approximately 40% of participants were younger than 50 years and 14.5% were younger than 40. Correspondingly, relevant clinical guidance allowing omission of nodal irradiation does not generally include an age restriction [33].

#### 3.4.6. Omission of Adjuvant Radiotherapy

For both DCIS and invasive breast cancer, data support that in selected low-risk patients, adjuvant radiotherapy may be omitted under specific conditions. Trials such as CALGB 9343 and PRIME II included older patients (≥70 and ≥65 years, respectively) and demonstrated that radiotherapy omission may be considered in elderly women after thorough counseling and informed consent, acknowledging the risk in general, that omission of radiotherapy is associated with an expected approximately 10% risk of locoregional recurrence (LRR) in 10 years [34,35]. This approach has been incorporated into ASTRO, NCCN, and ESMO guidelines, which specifically address older patients.

More recent studies incorporating genomic assays as predictive biomarkers have sought to identify low-risk patients in whom radiotherapy can be safely omitted. The IDEA trial, a single-arm study enrolling patients with pT1N0 disease, widely negative margins, the Luminal A subtype, and an Oncotype DX 21-gene recurrence score ≤18, omitted radiotherapy. After 5 years of follow-up, recurrence was only 1%. However, only postmenopausal patients were included, and therefore no conclusions can be drawn for younger women [36].

The recurrence score as a biomarker of risk, including local recurrence risk, remains of interest, and results are awaited from NRG-BR007, a phase III randomized trial comparing adjuvant radiotherapy versus omission in favorable-risk patients with an Oncotype DX recurrence score ≤18 [37].

Similarly, the LUMINA a single-arm trial used Ki-67 as a biomarker. In very low-risk Luminal A patients with Ki-67 ≤13.25%, adjuvant radiotherapy was omitted and patients received endocrine therapy alone. As in IDEA trial, local recurrence was very low, with only 2.3% experiencing recurrence at 5 years. However, both trials excluded premenopausal women (LUMINA minimum age >55 years), highlighting that these findings cannot be directly applied to younger patients, and evidence for RT omission in women <50 years remains lacking [38].

The question of the magnitude of the benefit of radiotherapy in patients with low-risk disease receiving effective systemic therapy remains open, and several ongoing trials aim to address it, including HERO trial. In this phase III randomized trial, patients with early-stage, node-negative, HER2-positive breast cancer treated with breast-conserving surgery (negative margins and sentinel lymph node biopsy or axillary dissection) who have received or will receive cytotoxic chemotherapy and HER2-targeted therapy will be randomized to standard breast radiotherapy plus continuation of trastuzumab to complete one year of treatment, versus trastuzumab alone. Notably, this study includes younger patients, enrolling women aged ≥40 years [39].

Therefore, as outlined above, there are currently no robust data supporting omission of radiotherapy in very young patients. At present, even in younger women with highly favorable disease profiles including low recurrence scores, adjuvant radiotherapy should still be employed.

A qualitative assessment of risk of bias, certainty of evidence, and applicability to younger patients for each radiotherapy de-escalation strategy is summarized in Table 3, while key characteristics of ongoing trials are summarized in Table 4.

## 4. Discussion

Radiotherapy de-escalation has become an established paradigm in the management of early breast cancer, supported by robust randomized evidence in predominantly older and postmenopausal populations. However, younger patients remain consistently underrepresented across pivotal trials, limiting the extrapolation of these strategies to women under 50 years of age and, in particular, to those under 40 (Table 2).

Younger age at diagnosis is strongly associated with adverse clinicopathological features, including higher grade, greater nodal involvement, unfavorable molecular subtypes, and an increased prevalence of germline BRCA mutations [5,6,7]. These characteristics constitute strong predictors of increased locoregional recurrence and inferior survival outcomes [38], questioning the safety of treatment de-escalation in this population. Consequently, chronological age remains a clinically relevant modifier of risk, even in the era of modern systemic therapy and molecular profiling. Prospective stratification of younger patients by molecular and germline risk in future trials is critical to ensure safe and effective application of de-escalation strategies.

Among de-escalation strategies, moderate hypofractionation (15–16 fractions) has the strongest evidence and can be considered safe irrespective of age. Randomized data, supported by long-term outcomes and reinforced by recent trials incorporating regional nodal irradiation, justify its routine use in younger patients [12,13,14,15,16,17]. In contrast, ultra-hypofractionation (five-fraction regimens), while attractive for reasons of convenience and resource utilization, lacks sufficient representation of very young women, and caution is warranted until age-specific long-term toxicity and efficacy data mature [18,19].

From a radiobiological perspective, breast cancer is generally considered to have a relatively low α/β ratio, estimated between 2 and 4 Gy, similar to that of late-responding normal tissues [40]. This similarity underpins the therapeutic rationale for hypofractionation: larger doses per fraction can be delivered without compromising tumor control while potentially reducing late toxicity. For example, using the linear–quadratic model, a schedule of 40 Gy in 15 fractions (2.67 Gy/fraction) yields a biologically effective dose (BED) for tumor control similar to 50 Gy in 25 fractions when assuming an α/β of 3–4 Gy, supporting the clinical equivalence demonstrated in randomized trials. However, while there is no definitive evidence that the intrinsic α/β ratio of normal breast tissue differs in younger individuals, younger patients have a longer post-treatment life expectancy, providing a greater window for late toxicities such as fibrosis, adverse cosmesis, and potential radiation-induced cardiac effects to develop and progress. Nonetheless, an analysis of 4 protocols including patients below 50 years of age with breast cancer receiving hypofractionated radiotherapy regimens reported excellent cosmesis at 5 years, assessed by both clinicians and patients [41]. Potential cardiac toxicity and in particular the development of ischemic heart disease remains a matter of concern. Data shows that major coronary events increase linearly by 7.4% per Gy of mean heart dose (95% CI, 2.9–14.5; *p* < 0.001), with no apparent threshold. The excess risk began within five years after radiotherapy and persisted for at least three decades, with similar proportional increases observed regardless of baseline cardiac risk factors [42]. However, it should be noted that predicting cardiac adverse events post-radiotherapy is still challenging [43]. The cumulative impact of fractionation, total dose, and heart dose constraints must be carefully considered. Consequently, while moderate hypofractionation (e.g., 15–16 fractions) has been robustly validated across age groups, caution is warranted when applying ultra-hypofractionation schedules to younger patients until long-term toxicity data mature. Dose constraints for organs at risk should be rigorously adhered to, and patient selection must consider individual risk factors for late toxicity, including age, breast size, and baseline cardiac risk. These radiobiological principles underscore the need for personalized approaches and further prospective evaluation in younger cohorts.

With respect to field de-escalation, the evidence supporting partial breast irradiation is largely confined to patients aged ≥45–50 years [26,27]. Current data do not adequately address the higher propensity for multicentric disease and biologically aggressive behavior in younger patients, and existing guidelines appropriately restrict PBI to older age groups [25,26,27,28,29,30]. In addition, in younger patients, several technical factors may influence outcomes with PBI. Target delineation can be challenging in smaller or denser breasts, making surgical-clip-based localization essential to accurately define the tumor bed. Margin size is particularly critical, as narrower margins may increase recurrence risk in this population. Dosimetric sparing of non-target breast tissue varies by modality: brachytherapy provides a highly localized dose but may result in dose heterogeneity, whereas external-beam approaches allow more uniform coverage but require careful planning to minimize the dose to adjacent organs. These technical nuances, along with the absence of supporting evidence, make the routine implementation of PBI in younger patients inadvisable until further dedicated research is available [3]. Advanced imaging, including MRI [44], and genomic profiling may help refine patient selection and reduce the risk of elsewhere failures, but these strategies remain largely untested in younger women and should be considered as hypothesis-generating in future studies.

Similarly, patients achieving complete nodal response following neoadjuvant chemotherapy may be considered candidates for omission of postmastectomy radiotherapy and/or regional nodal irradiation, but such an approach should be discussed case by case and the clinical decision should be mainly based on extent of pathological response and tumor biology rather than age alone [31,32].

Complete omission of adjuvant radiotherapy remains unsupported in very young patients, even in the presence of favorable clinicopathological features or low-risk genomic signatures [34,35,36,38,39]. Trials employing biomarkers such as Oncotype DX or Ki-67 have systematically excluded premenopausal or younger women, leaving a critical evidence gap [36,38,39]. Until ongoing randomized studies specifically address this population, radiotherapy omission should not be routinely offered to younger patients outside clinical trials.

It should be noted that interpretation of radiotherapy de-escalation trials in younger patients must account for important methodological limitations. Most randomized studies were open-label by design, which may influence reporting of subjective toxicity endpoints. In addition, younger patients were frequently underrepresented or excluded altogether, limiting the directness of evidence. Selection criteria often favored patients with small tumors, favorable molecular subtypes, and low recurrence risk, while imbalances and evolution in systemic therapy across trials further complicate extrapolation to contemporary younger cohorts. These factors collectively reduce the certainty of evidence for aggressive de-escalation strategies in younger women, despite robust overall results in older populations.

Further focusing on the issue of systemic therapy, it should be considered that many of the current systemic treatments and newer targeted agents were not available at the enrollment period of the reported studies. The routine use of taxane-based chemotherapy regimens, peri-operative chemo-imunotherapy HER2-targeted agents such as trastuzumab, adjuvant CDK 4/6 inhibitors and extended endocrine therapy durations have significantly improved systemic control and reduced distant and locoregional recurrence risks [45]. Additionally, extended axillary lymph node dissection has been replaced by sentinel lymph node biopsy in many cases and enhanced imaging modalities and clinical staging have refined patient selection and risk stratification [40]. These changes may bias locoregional outcomes observed in historic trials, which often included patients treated with less effective systemic therapy and less precise staging. Consequently, the external validity of older radiotherapy de-escalation trials for contemporary younger patients, who typically receive multimodal, optimized treatment, may be limited. This emphasizes the critical need for modern prospective studies incorporating current systemic treatments and diagnostic approaches to more accurately define safe radiotherapy de-escalation strategies in this population.

Importantly, long-term toxicity data specific to younger breast cancer patients remain limited. While some trials include younger cohorts, mature late toxicity endpoints (≥5–10 years) stratified by age are rarely reported separately, often resulting in pooled analyses with limited granularity. Consequently, conclusions regarding safety and late effects in younger patients must be interpreted with caution and are inherently of low certainty. This underscores the critical need for dedicated, long-term follow-up focusing on younger populations to better characterize late toxicities such as fibrosis, cosmesis changes, and cardiac sequelae. Until such data mature, clinicians should exercise prudence when extrapolating existing toxicity outcomes to younger women and advocate for prospective trials with robust age-specific toxicity reporting.

Finally, based on current evidence summarized in Table 2, radiotherapy de-escalation strategies must be interpreted with caution in younger patients. For women <40 years, moderate hypofractionation with or without regional nodal irradiation is considered standard of care, whereas ultra-hypofractionation, partial breast irradiation, RT omission, and biomarker-guided strategies remain investigational. Evidence in this age group is limited, follow-up is short, and long-term toxicity data are sparse. For women 40–49 years, moderate hypofractionation (with or without nodal irradiation) is standard, ultra-hypofractionation can be considered with caution, and PBI or RT omission strategies should generally be avoided outside of trials. For women ≥50 years, moderate hypofractionation, selected PBI, and omission strategies in low-risk patients are supported by robust evidence. Across all ages, genomic profiling and advanced imaging may help refine patient selection and reduce the risk of elsewhere failures in the breast, but their integration into routine practice remains investigational.

## 5. Conclusions

Radiotherapy de-escalation in younger patients with breast cancer must be approached with caution. While moderate hypofractionation is now clearly supported regardless of age, more aggressive de-escalation strategies—including ultra-hypofractionation, partial breast irradiation, and omission of radiotherapy—lack robust evidence in women under 50 years, particularly those under 40. Considering the distinct biological and clinical behavior of breast cancer in younger patients, age remains an essential parameter in radiotherapy decision-making. Future trials with adequate representation of younger women, stratified by molecular subtype and genomic risk, are required to clarify whether de-escalation paradigms can be safely extended to this population.

## Figures and Tables

**Table 1:** Hypofractionated Breast Radiotherapy: Age Eligibility and Representation in Key Trials.

Trial	Fractionation Schedule	Minimum Eligible Age	Median/Mean Age	<50	<40	Key Age-Related Findings
Canadian Trial [17]	42.5 Gy/16 fx vs. 50 Gy/25 fx	≥18 years	50	~30%	~7%	No interaction between age and local control or toxicity
START A [12]	41.6 Gy/13 fx or 39 Gy/13 fx vs. 50 Gy/25 fx	≥18 years	57	~26%	~6%	Age not predictive of recurrence or late effects
START B [13]	40 Gy/15 fx vs. 50 Gy/25 fx	≥18 years	57	~24%	~5–6%	Equivalent local control across age groups
MD Anderson [16]	42.56 Gy/16 fx vs. 50 Gy/25 fx	≥18 years	51	~35%	~8%	Hypofractionation superior for acute toxicity, independent of age
FAST [18]	28.5 Gy or 30 Gy/5 fx (weekly) vs. 50 Gy/25 fx	≥50 years	62	0%	0%	Younger patients excluded
FAST-Forward [19]	26 Gy or 27 Gy/5 fx vs. 40 Gy/15 fx	≥18 years	61	15% (40–49)	~2%	Limited data in <40; non-inferior local control overall
HYPOG-01 [20]	40 Gy/15 fx vs. 50 Gy/25 fx (incl. nodal RT)	≥18 years	53	Not reported	Included	Non-inferiority maintained across age groups

**Table 2:** Radiotherapy De-escalation Strategies by Age Group in Breast Cancer.

De-Escalation Strategy	Age Group			Key Evidence; Comments
	<40 Years	40–49 Years	≥50 Years	
Moderate hypofractionation (15–16 fractions)	Yes; Standard of care	Yes; Standard of care	Yes; Standard of care	START A/B, Canadian, MD Anderson; no age–treatment interaction observed
Moderate hypofractionation with regional nodal irradiation	Yes; Standard of care	Yes; Standard of care	Yes; Standard of care	HYPOG-01 demonstrated non-inferiority; younger patients included
Ultra-hypofractionation (5 fractions)	Uncertain	Acceptable with caution	Yes; Supported evidence	FAST-Forward included very few <40 (≈2%); long-term toxicity data limited in young women
Partial breast irradiation (PBI)	No	Generally No (selected ≥45)	Yes; Selected low-risk patients	Florence, IMPORT LOW; most trials restricted to ≥45–50
Omission of chest wall RT after mastectomy (intermediate risk)	Uncertain	Uncertain	Possible in selected patients	SUPREMO included younger patients but current guidelines remain conservative
Omission of nodal RT after nodal pCR to NAC	Yes	Yes	Yes	NSABP B-51 included substantial <50 and <40 cohorts; age not a limiting factor
Omission of RT in invasive breast cancer	No	No	Yes; Selected elderly only	CALGB 9343, PRIME II restricted to ≥65–70
Genomic assay-guided RT omission (Oncotype DX, Ki-67)	No	No	Investigational; Selected low-risk patients	IDEA, LUMINA excluded premenopausal women; NRG-BR007 trial ongoing
RT omission in HER2-positive disease	Investigational	Investigational	Investigational	HERO trial ongoing includes ≥40

**Table 3:** Risk of Bias, Certainty of Evidence, and Applicability to Younger Patients by De-escalation Strategy.

De-Escalation Strategy	Key Trials	Major Risk of Bias/Limitations	Certainty of Evidence (GRADE-Style)	Applicability to Younger Patients
Moderate hypofractionation (15–16 fx)	START A/B, Canadian, MD Anderson, HYPOG-01	Open-label design; limited <40 subgroup; heterogeneity in boost and systemic therapy	High	High—consistent outcomes, no age–treatment interaction
Ultra-hypofractionation (5 fx)	FAST, FAST-Forward	Older, low-risk populations; very small <40 cohort; limited long-term toxicity data	Moderate	Low–Moderate—caution in very young patients
Partial breast irradiation (PBI)	Florence, IMPORT LOW, GEC-ESTRO	Restrictive age criteria; selection of favorable biology; exclusion of high-grade disease	Moderate	Low—insufficient evidence <45–50
Omission of radiotherapy	CALGB 9343, PRIME II, IDEA, LUMINA	Elderly/postmenopausal cohorts; biomarker-selected populations; non-randomized designs	Low	Very Low—not supported in young patients

**Table 4:** Ongoing De-escalation Trials.

Trial Name	ClinicalTrials.gov ID	Target Enrollment	Age Eligibility	Primary Endpoint(s)	Estimated Primary Completion	Notes
HERO/NRG-BR008	NCT05705401	~1300	≥40 years	Recurrence-Free Interval (RFI)	2034 (Primary Completion)	Omission of RT vs. standard for low-risk HER2+; assesses RFI, ipsilateral recurrence & quality of life
NRG-BR007/DEBRA	NCT04852887	~1670	50–70 years	Ipsilateral Breast Tumor Recurrence (IBTR); relapse-free interval	~2026	Omission vs. standard RT + endocrine therapy in low-risk HR+/HER2-; endocrine therapy alone experimental arm
PRECISION	NCT02653755	~672	50–75 years	Locoregional Recurrence	~2026	Biomarker-directed omission trial using PAM50 ROR score
PRIMETIME	ISRCTN41579286	~2400	≥60 years	Ipsilateral Breast Tumour Recurrence (5 yrs)	~2027	Biomarker-directed omission using IHC4 + C
EXPERT	(various Regs)	~1167	≥50 years	Locoregional Recurrence	~2024+	Uses PAM50; radiotherapy omission vs. PBI/standard as applicable

## Data Availability

No new data were created or analysed in this study. Data sharing is not applicable to this article.

## Supplementary Materials

The following supporting information can be downloaded at: https://www.mdpi.com/article/10.3390/cancers18040639/s1, File S1: PRISMA 2020 Checklist.
