# Supplementary material for: Radiotherapy De-Escalation in Younger Patients with Breast Cancer: Are We There Yet?
Source: Cancers (Basel). 2026 Feb 16;18(4):639. doi: 10.3390/cancers18040639 (PMC12939668; doi:10.3390/cancers18040639)
Supplement: Supplementary file 1 [file cancers-18-00639-s001.zip › cancers-4093074-supplementary.pdf]

PRISMA 2020 Checklist

Manuscript title: Radiotherapy De-escalation in Younger Patients with Breast Cancer: Are We There Yet?
Journal: Cancers (MDPI)

This checklist is completed in accordance with the PRISMA 2020 Statement.

Section / Topic	Item No.	PRISMA 2020 Checklist Item	Location in Manuscript
Title	1	Identify the report as a systematic review.	Title page
Abstract	2	Provide a structured summary.	Abstract
Introduction	3	Describe the rationale for the review.	Introduction
Objectives	4	Provide an explicit statement of objectives or questions.	Introduction
Eligibility criteria	5	Specify inclusion and exclusion criteria.	Materials and Methods
Information sources	6	Specify all databases, registers, and other sources searched.	Materials and Methods
Search strategy	7	Present the full search strategies for all databases.	Materials and Methods
Selection process	8	Describe the process for selecting studies.	Materials and Methods
Data collection	9	Describe how data	Materials and

process		were collected.	Methods
Data items	10a	List and define all outcomes and other variables.	Materials and Methods
Risk of bias	11	Specify methods used to assess risk of bias.	Materials and Methods
Effect measures	12	Specify effect measures used.	Not applicable (qualitative synthesis)
Study selection	16a	Describe results of the search and selection process.	Results; Figure 1
Study characteristics	17	Cite each included study and present characteristics.	Results; Tables 1–4
Risk of bias in studies	18	Present risk of bias assessments.	Results; Table 3
Results of individual studies	19	Present summary statistics for each study.	Results
Results of syntheses	20	Present results of all syntheses.	Results
Reporting bias	21	Assess risk of reporting bias.	Discussion
Certainty of evidence	22	Assess certainty or confidence in the body of evidence.	Results; Table 3
Discussion	23	Provide a general interpretation of results.	Discussion
Limitations	23b	Discuss limitations of the evidence and review process.	Discussion

Conclusions	23c	Provide implications for practice and future research.	Discussion; Conclusions
Registration	24a	Provide registration information.	Materials and Methods
Protocol	24b	Indicate where the protocol can be accessed.	Materials and Methods
Support	25	Describe sources of support.	Funding
Competing interests	26	Declare competing interests.	Conflicts of Interest
Availability of data	27	Report availability of data and materials.	Data Availability Statement
